# Misconception between palliative care and euthanasia among Thai general practitioners: a cross-sectional study

**DOI:** 10.1186/s12904-024-01430-6

**Published:** 2024-04-11

**Authors:** Lalita Chutarattanakul, Viriya Jarusukthavorn, Nisachol Dejkriengkraikul, Myo Zin Oo, Soe Sandi Tint, Chaisiri Angkurawaranon, Nutchar Wiwatkunupakarn

**Affiliations:** 1 Department of Family Medicine, Faculty of Medicine, Chiang Mai University, 110 Intawaroros Road, Si Phum, Muang, Chiang Mai, 50200 Thailand; 2 Global Health and Chronic Conditions Research Center, Chiang Mai University, Chiang Mai, Thailand

**Keywords:** Attitude, Confidence, Euthanasia, Knowledge, Misconception, Palliative care, Practical experience

## Abstract

**Background:**

Palliative care lower medical expenses and enhances quality of life, but misconception with euthanasia delays timely care and makes inappropriate patient management.

**Objective:**

To examine the magnitude of misconceptions between palliative care and euthanasia among Thai general practitioners, explore the association with knowledge, attitudes, and practical experience, and assess the association between misconception and confidence in practicing and referring patients to palliative care centers.

**Methods:**

All 144 general practitioners who were going to start residency training at Maharaj Nakorn Chiang Mai Hospital in 2021 participated in this observational cross-sectional study. A chi-square test was utilized to examine the relationship between misconception and knowledge, attitude, practical experience, confidence to practice, and confidence to refer patients. Multivariable logistic regression was carried out while controlling for age, sex, knowledge, attitude, and experience to examine the relationship between misconception and confidence to practice and refer patients for palliative care. Statistical significance was defined at *p* < 0.05.

**Results:**

About 41% of general physicians had misconceptions regarding palliative care and euthanasia. High knowledge was associated with a lower level of misconception (*p* = 0.01). The absence of misconceptions was weakly associated with a higher level of confidence in practicing palliative care, with an adjusted odds ratio of 1.51 (95% confidence interval 0.73 to 3.10, *p* = 0.07).

**Conclusion:**

High misconception rates between palliative care and euthanasia among young Thai physicians might impact their confidence in delivering palliative care. Training initiatives for medical students and practitioners can mitigate misconceptions, fostering better palliative care utilization in Thailand.

**Supplementary Information:**

The online version contains supplementary material available at 10.1186/s12904-024-01430-6.

## Background

Palliative care (PC) provides specialized medical care and support to improve patients’ quality of life and reduce healthcare costs [1–5]. The primary goal of PC is to provide relief from symptoms such as pain, nausea, and shortness of breath and to address the physical, emotional, social, and spiritual needs of patients, which involves multiple actors and teamwork to deliver coordinated and integrative care [2, 6]. Previous study has shown that this service can save costs by reducing hospital readmissions, avoiding unnecessary treatments, and improving the efficient use of healthcare resources [7]. As a result, the demand for PC has increased during the last decade [8].

The ideal PC approach should be started at the early stage of the patient’s illness trajectory (e.g., after diagnosis of incurable conditions or after the disease begins to get worse), especially in patients with advanced cancer [9]. Early PC consultation provides more opportunities for patients and providers to prepare for symptom and emotional management when the last day comes [10, 11]. It was found that early PC consultation is associated with a lower length of hospital stay after consultation, fewer inpatient deaths, and higher hospice admission instead of long-term acute care [12].

However, the PC approach, particularly during end-of-life care, is often misunderstood as ‘euthanasia.‘ [13] People frequently inaccurately associated PC with patients losing hope, perceived as a period of waiting for death when all treatment options have been exhausted [14]. The prevalence of misconceptions about these terms in healthcare professionals ranged between 2–56%, depending on the topics [15], reflecting a significant need for education and awareness regarding PC’s true nature and benefits.

Misconception is defined as ideas that are inconsistent with scientific knowledge and result in misunderstanding and misinterpretation [16]. In this study, we examined ideas that are inconsistent with scientific knowledge about PC and euthanasia and the subsequent potential decrease in confidence in practising and referring patients for PC. Euthanasia is the act of intentionally ending a person’s life to relieve their suffering [17]. It is typically administered by a healthcare professional or another person at the patient’s request. Euthanasia can be either voluntary (with the explicit consent of the patient) or involuntary (without the patient’s consent, which is illegal in many places) [18], and either active (taking a direct action to end a patient’s life, such as administering a lethal injection) and passive (withholding or withdrawing life-sustaining treatments with the expectation that it will lead to the patient’s death) [19].

Physicians may occasionally misunderstand the difference between PC and euthanasia because of a lack of education and training, complex ethical and legal frameworks, and cultural and personal beliefs [20–23]. Medical professionals may not receive adequate education and training in end-of-life care, including PC principles, which results in a limited understanding of the distinctions between these terms. The ethical and legal landscape surrounding end-of-life care can be complex and vary widely by region, making it challenging for physicians to navigate these complexities. Finally, cultural, religious, or personal beliefs can influence a physician’s perspective on end-of-life care, which leads to differing interpretations of what constitutes appropriate care in a given situation.

In Thailand, PC needs have been rising due to the aging society [24–26]. The government is running political actions to promote PC in primary care settings, including caring for patients at home, hospitals, and hospices [25]. The number of medical schools opening PC-related curricula (e.g., PC fellowship and residency) or integrating PC knowledge into medical student programs is increasing to provide appropriate PC in Thailand. Around 2,000 medical students graduate as GP yearly. Most new graduates will spend up to three years as a GP in general hospitals before returning for further specialisation. In these first three years, they will need to care for common conditions in community hospitals, both in-patient and out. These tasks will include caring for terminally ill patients at the hospital, the patient’s home, or nursing home [27].

While PC is widely accepted, euthanasia is not legally sanctioned. Assisting or participating in euthanasia can lead to criminal charges in the country. Thus, investigating the magnitude of misconception between PC and euthanasia is important in terms of evaluating the provider’s understanding and the appropriateness of the training programs.

Misconceptions are associated with several factors. It is influenced by providers’ knowledge, attitude, and practical experience (KAP) [28–31]. A lack of knowledge, negative attitude, and insufficient professionalism in healthcare providers during the management of end-of-life in terminally ill patients can contribute to both medical and ethical problems. Misconception may also affect doctors’ performance, leading to low confidence in delivering PC and referring patients to PC centers [32]. This consequence could contribute to the delay of treatment and inappropriate management of PC patients.

**Fig. 1:**
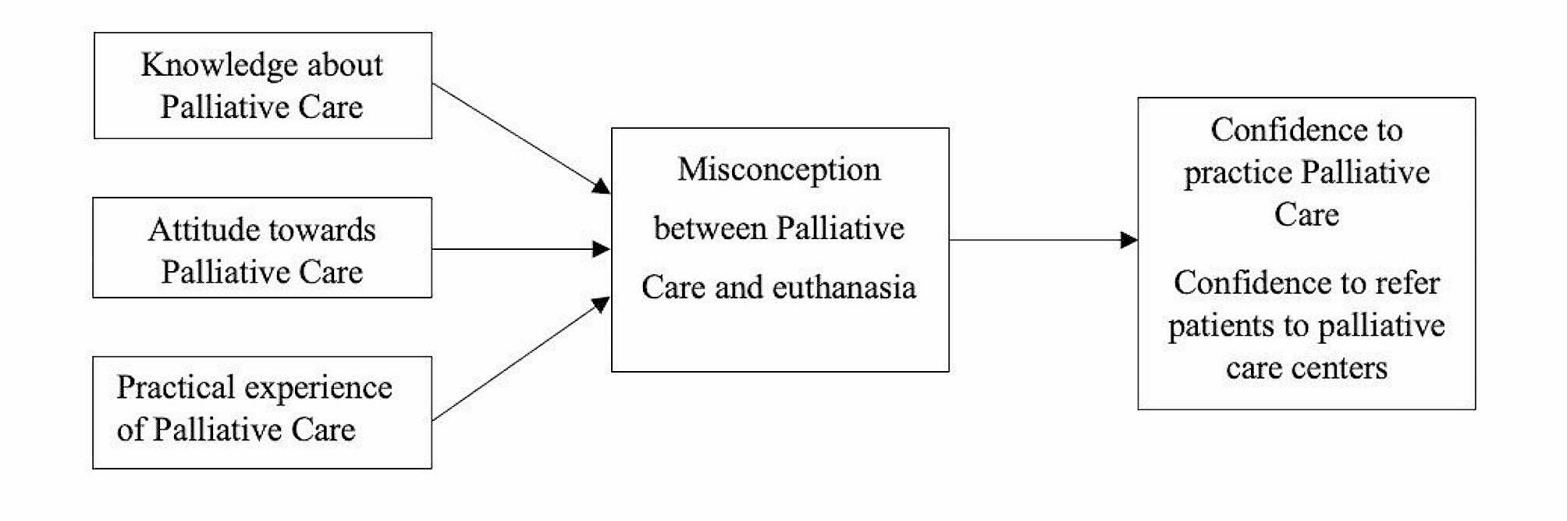
Conceptual framework of the study

Therefore, the primary objective of this study is to explore the magnitude and details of the misconception between PC and Euthanasia among Thai general practitioners. The secondary objectives are (1) to investigate the association between knowledge, attitude, practical experience, and misconception and (2) to investigate the association between misconception and confidence to practice PC and refer patients to PC centers. The conceptual framework of this study is shown in Fig. 1.

## Methods

### Study design, setting, and participants

A cross-sectional study was conducted among all 144 general practitioners about to start residency training at Maharaj Nakorn Chiang Mai Hospital in 2021. Maharaj Nakorn Chiang Mai Hospital is a university hospital in Chiang Mai province (the center of northern Thailand) providing care and treatment in many specialties. Researchers recruited all these general practitioners on the first day of orientation before they started their residency training.

### Sample size

According to the previous literature, the estimated percentage of healthcare professionals who misunderstand PC and euthanasia is 17.1% [15]. Using sample size calculation for one proportion [33], with an estimated error of 0.1, at least 55 participants are required for the survey.

### Measurements

A self-administered, structured questionnaire was developed for this study by applying different sources of evidence (Supplementary File 1). The questionnaire consisted of the following sections:

#### Participants’ background

This section covered age, sex, years of working experience after graduation, PC topics studied during Medical School, and additional training on PC after graduation.

#### Misconception between PC and euthanasia

This section included a total of 8 statements about palliative care or euthanasia. For each statement, participants had to identify whether the statement was true or false. Five statements were about PC principles characterized by The European Association of Palliative Care (EAPC) regarding palliative sedation, living will, opioids used to relieve intolerable symptoms, and withholding/withdrawing life-sustaining treatment [34–37]. Three statements were about euthanasia, regarding intentionally ending a person’s life under the administration of a physician by using medication [35, 38, 39]. The questionnaire was piloted in several GPs and checked for its content-related validity by 2 PC experts before being used in this study. Responses from participants were scored, with correct responses receiving one point and wrong ones obtaining zero points. Scores ranged from 0 to 8, with a score of 8 indicating no misconceptions (between palliative care and euthanasia) and less than eight suggesting misconceptions.

#### Knowledge about PC

This section included a total of 16 multiple choice questions (one best answer) regarding the delivery of bad news, various cancer treatment modalities, appropriate reasons for referring patients to hospice, cancer pain management, different types of opioids, non-pain management, management in final days, emergency symptoms management and psychological care. The questions were adopted from a prior Thai study by Budkaew and Chumworathayi [40]. Additional contents on living will and advanced care planning were also added. Scores ranged from 0 to 16, with the threshold between low and high levels of knowledge defined by the interquartile range (IQR).

#### Attitude towards PC

This section included 22 questions regarding attitudes toward PC, such as the importance of PC and PC planning. These attitude questions were adopted from the study by Budkaew and Chumworathayi [40]. A 5-point Likert scale ranged from ‘strongly disagree’ [1] to ‘strongly agree’ [5]. Total attitude scores ranged from 22 to 110, and the IQR determined the threshold between low and high levels of (positive) attitude towards PC.

#### Practical experience with PC

The set of questions about practical experience was developed to ascertain participants’ experience in caring for PC patients throughout their careers by asking about the number of PC cases they have encountered. The responses were categorized into three categories: 0, 1–9, and 10 cases and over.

#### Level of confidence to practice PC and to refer patients to PC center

Lastly, confidence in performing PC was assessed across two aspects: caring for patients with palliative conditions and referring patients to PC centers. Each aspect of confidence was rated from 0 to 10, with higher scores indicating greater confidence. The median score was determined, with scores more than the median representing a high confidence level and scores less than the median representing a low level of attitude.

### Data analysis

The data analysis was conducted using SPSS version 25. For socio-demographic characteristics, misconceptions, KAP scores, confidence to practice, and confidence to refer patients were analyzed using frequency and percentage for categorical data and mean with its standard deviation for continuous data. The chi-square test was used to test the association between knowledge, attitude, practical experience, confidence to practice, confidence to refer patients, and misconception. Multivariable logistic regression was performed to investigate the association between misconception and confidence to practice and refer patients for PC, adjusting for age, sex, knowledge, attitude, and experience. Statistically significant associations were set as those with a *p*-value less than 0.05.

## Results

### Participants’ background

One hundred and forty-four general practitioners participated in this study. The mean age was 26.4 years, which ranged between 23 and 38 years. The proportions between men and women were similar. On average, participants had worked for 1.77 years, with 48.6% working for one year or less. Most of these participants (*N* = 137/144) studied at least one topic about PC when they were medical students. During medical school, the majority were taught about pain management (84.7%), advance care planning (74.5%), living will (65.7%), end-of-life care (59.1%), and PC systems (54.0%). However, less than half were taught about family and caregiver support (43.8%) and other symptom management (43.8%). After graduation, only 27.1% had undergone any additional palliative care training (See Table 1).

**Table 1:** Participants’ background

Socio-demographic and Professional characteristics (*N* = 144)	Frequency (N)	Percentage (%)
**Age**		
≤ 25	66	45.8
26–35	72	50.0
≥ 36	6	4.2
Mean ± SD (Min-Max)	26.39 ± 2.91 (23–38)	
**Gender**		
Male	74	51.4
Female	70	48.6
**Working years after graduated from medical school**		
≤ 1 year	70	48.6
> 1 year	74	51.4
Mean ± SD (Min-Max)	1.77 ± 1.94 (0–13)	
Palliative care studies at the undergraduate level* ( ***N*** ** = 137)**		
End of life care	81	59.1
Advance care planning	102	74.5
Living will	90	65.7
Pain management	116	84.7
Other symptom management	60	43.8
PC system	74	54.0
Family and care giver support	60	43.8
**Additional training on PC after graduation**		
No	105	72.9
Yes	39	27.1
Frequency of training** ( ***N*** ** = 39)**		
1	26	66.6
2	12	30.8
3	1	2.6

*Multiple select question

** For those who attended additional training on PC

### Misconceptions between palliative care and euthanasia

41% of participants had at least one misconception between PC and Euthanasia. The questions that were frequently answered incorrectly pertained to the misunderstanding that following patients’ living will by withdrawing endotracheal intubation is euthanasia (16.7%) and using sedative drugs or muscle relaxants to hasten the end of a patient’s life is PC (16.0%). Additionally, there were misconceptions surrounding allowing patients to die at home as wished is euthanasia (12.5%), and employing sedative drugs for symptom control is euthanasia (12.5%). However, most physicians answered questions correctly regarding morphine use to hasten death as euthanasia and relieving respiratory distress is PC (See Table 2).

**Table 2:** Detail of misconception between PC and euthanasia

Misconception (*N* = 144)	Correct	Incorrect
	Frequency (Percentage)	Frequency (Percentage)
“Using a sedating drug in those imminently dying intention to relieve intolerable suffering by controlling the symptoms and Intentional administration of sedatives to reduce a dying person’s consciousness to relieve in tolerable suffering from refractory symptoms” is palliative care (true)	125 (87.5)	18 (12.5)
“Titration of morphine in terminal cancer patient for relieving respiratory distress” is palliative care (true)	140 (97.2)	4 (2.8)
“Intravenous infusion of morphine overdose in order to end the sufferer’s life” is euthanasia (true)	141 (97.9)	3 (2.1)
“Physician refuses to insert endotracheal tube (intubation) because the physician wants to follow patient’s living will” is palliative care (true)	120 (83.3)	24 (16.7)
“Physician allows the relatives to take the terminal cancer patient back to dying at home as patient’s wishes” is palliative care (true)	126 (87.5)	18 (12.5)
“Physician intentionally ends patient’s life by the administration of sedative drug or muscle relaxant” is euthanasia (true)	121 (84.0)	23 (16.0)
“Lung cancer stage 4 patient, who had completed operation, chemotherapy and radiation but the disease’s still progressive. Physician does family meeting about discontinue the chemotherapy and treat as palliative caring instead” is palliative care (true)	141 (97.9)	3 (2.1)
“Physician administrates anesthesia in order to hasten patient’s death, as patient’s competent request because of his incurable disease” is euthanasia (true)	135 (93.8)	9 (6.2)

### Relationships between KAP and misconceptions

Both higher knowledge and attitude were linked to the absence of misconceptions, yet statistical significance was solely observed in the association between knowledge and misconception. Having high knowledge of PC was associated with lower proportions of misconceptions (*p* = 0.01). 70% of the total participants had experience in palliative care work. But only 13.9% had cared for more than 10 cases. The proportion with misconception varied by prior practical experience, but the observed differences were not statistically significant (*p*-value = 0.674) (See Table 3).

**Table 3:** Association between knowledge, attitude, practical experience, confidence, and misconception

Variable	Total Frequency (Percentage)	Misconception		*P* value
		Yes (*N* = 59)	No (*N* = 85)	
		Frequency (Percentage)	Frequency (Percentage)	
**Knowledge**				
Low	53 (36.8)	29 (49.2)	24 (28.2)	0.010*
High	91 (63.2)	30 (50.8)	61 (71.8)	
**Attitude**				
Low	39 (27.1)	20 (33.9)	19 (22.3)	0.125
High	105 (72.9)	39 (66.1)	66 (77.7)	
**Practice**				
Number of treated terminally ill patient in career life				
0	42 (29.2)	19 (32.2)	23 (27.1)	0.674
1–9	82 (56.9)	31 (52.5)	51 (60.0)	
≥ 10	20 (13.9)	9 (15.3)	11 (12.9)	
**Level of confidence**				
Confidence to practice				
Low	74 (51.4)	33 (55.9)	41 (48.2)	0.244
High	70 (48.6)	26 (44.1)	44 (51.8)	
Confidence to refer patients				
Low	74 (51.4)	34 (57.6)	40 (47.1)	0.363
High	70 (48.6)	25 (42.4)	45 (52.9)	

### Relationships between misconceptions and level of confidence to practice and refer patients to PC center

The absence of misconception was related to a higher level of confidence in both practicing and referring; however, only weak evidence of an association between misconception and confidence to practice was shown (confidence to practice: adjusted OR 1.15, 95% CI 0.73–3.10, *p*-value = 0.068; confidence to refer: adjusted OR 1.51, 95% CI 0.75–3.03, *p*-value = 0.773) (See Table 4).

**Table 4:** Association between misconception and confidence to practice PC and refer patient for PC

Misconception	Confidence (Practice)				Confidence (Refer)			
	Crude OR (95% CI)	*p*-value	Adjusted OR (95% CI)	*p*-value	Crude OR (95% CI)	*p*-value	Adjusted OR (95% CI)	*p*-value
Yes	1 (ref)	0.363	1 (ref)	0.068	1 (ref)	0.212	1 (ref)	0.773
No	1.36 (0.69–2.65)		1.51 (0.73–3.10)		1.53 (0.78–2.99)		1.51 (0.75–3.03)	

* Adjusted with age, sex, knowledge, attitude, work experience and number of treated terminally ill patient

## Discussion

This study found that the prevalence of misconception among general practices is common. Many physicians misunderstood that withdrawing life-sustaining procedures as indicated in the patient’s living will and discharging palliative patients to die at home are part of euthanasia. At the same time, some are confused that using sedative drugs and muscle relaxants to fasten patients’ death is palliative care, but using them to relieve suffering is euthanasia. High knowledge, a high positive attitude, and high practical experience were associated with a lower risk of misconception. However, a significant difference was only observed in the association of misconception with knowledge. The absence of misconceptions was weakly associated with higher confidence to practice PC.

To our knowledge, this is the first study investigating among physicians the magnitude of misconception between PC and Euthanasia in Thailand. Most participants were taught about pain management and advanced care planning in undergraduate courses, but other topics were less likely to be taught. Therefore, physicians answered questions about morphine use and family meeting more correctly than the others. These findings corresponded with a study among Canadian physicians, which found that symptom management and communication skills are the competency areas readily taught, and what we need now is a program tailored to practice contexts [41]. Our findings suggest that further development of undergraduate PC courses should focus more on other topics, such as the contents of discontinuing unnecessary medical procedures and utilizing sedative drugs and muscle relaxants in terminally ill patients.

This study investigated the relationship between misconception and its contributing factors: knowledge, attitude, and practice/experience. Previous studies showed that knowledge, attitude, and misconception interact with each other, as good knowledge and a positive attitude promote a better understanding of the concept [1] and a low level of misconception was associated with a more positive attitude [42]. Therefore, building a good understanding of PC among GPs requires knowledge improvement and positive attitude development. Our study showed a similar trend as physicians with high knowledge and positive attitudes had a lower percentage of misconception. However, even with a large proportion of those with good knowledge and attitude, the percentage of misconception is still high. This might be due to another contributing factor, such as practical experience [28–31].

More than half of the surveyed physicians in this study performed PC. However, most of them managed fewer than 10 cases throughout their careers. This trend can be attributable to their relatively new status as medical practitioners, as the participants’ average age was 26.4 years, and Thai medical students typically complete their education by the age of 24–25 years. Moreover, as there is no formal training in PC for undergraduate students in Thailand, it is less likely for medical students to have experience in PC management, only being lectured in the classroom. A lack of adequate practical experience may affect students’ understanding of the concept of PC in depth. Previous study revealed that enhanced practical experiences strengthen understanding, theoretical knowledge, and concepts, attributed to clinical exposure [43–45]. Therefore, the evidence in our study suggested that further PC training for both undergraduate and postgraduate medical students should provide more hours for them to manage terminally ill patients, as training is crucial to enhance one’s knowledge, attitude, and practical experience when performing medical tasks [46–48].

Finally, our study supports that misconception was weakly associated with lower confidence to deliver care. They may experience ethical and moral dilemmas when they believe they are being asked to participate in or provide euthanasia, even when they are actually providing palliative care [49, 50]. They may fear legal repercussions for providing palliative care that is incorrectly perceived as euthanasia [51]. These dilemmas and fears can create internal conflicts and reduce their confidence in their ability to provide appropriate care [52]. Our study showed those who misunderstood were more likely to have low confidence. Previous study also demonstrated similar finding on the relationship between misconception and the level of confidence to deliver care [53, 54]. However, many factors are involved in this association [55], and further studies exploring the causal relationship between these variables are suggested.

Therefore, based on the KAP framework and findings in this study, we suggest improving GPs’ knowledge and practical experience. Undergraduate curricula should include topics related to (1) discontinuation of unnecessary medical procedures and (2) management of sedatives, analgesics, and muscle relaxants in PC patients. Case-based learning and short didactic lectures have demonstrated the potential to expand and distribute knowledge and skills in the field of PC [56]. They have significantly increased physicians’ confidence in practising PC in the Thai context [57].

### Limitations

First, the sample size was small, which resulted in low power; thus, some findings were only marginally significant. We recruited general practitioners from only one site. Further study should be conducted to improve generalizability. Lastly, there will be limitations regarding causal relationships between variables due to the cross-sectional design. However, there are also some strengths to our study. It is one of the first studies that examines the extent of misconception between PC and euthanasia among physicians in Thailand, and results could be useful in other settings where formal training in palliative care is in its early stages. Additionally, the tool for evaluating misconceptions between PC and euthanasia was developed from international definitions and standards. The findings of this study should have implications for educational interventions, policy formulation, and professional training programs aiming at improving physicians’ understanding of PC and associated ethical issues.

## Conclusion

This study highlights the high prevalence of misconception between palliative care and euthanasia among young Thai physicians, which might affect their confidence in delivering palliative care in practice. Providing medical students and general practitioners with training and additional opportunities to handle palliative care patients may be useful in reducing misconceptions between palliative care and euthanasia and promoting the utilization of palliative care in Thailand.

### Electronic supplementary material

Below is the link to the electronic supplementary material.

Supplementary Material 1

## Data Availability

The data supporting the findings in this manuscript are available upon reasonable request to the corresponding author due to ethical agreement on data privacy.

## Abbreviations

PC: Palliative Care
