# Supplementary material for: Misconception between palliative care and euthanasia among Thai general practitioners: a cross-sectional study
Source: BMC Palliat Care. 2024 Apr 11;23:96. doi: 10.1186/s12904-024-01430-6 (PMC11007896; doi:10.1186/s12904-024-01430-6)
Supplement: Supplementary file 1 — Supplementary Material 1 [file 12904_2024_1430_MOESM1_ESM.docx]

**Questionnaire**

**Part 1 General Information ( 5 items )**

Direction: Please answer the questionnaire about yourself by ticking ( √ ) in front of the corresponded answer or fill in the blank.

1. Gender

- Male
- Female

2. Age ………………………….. years

- Other fields, please specify ................................................ .......

3. Experience of working in a hospital after graduation………. years

4. While studying for a Bachelor's degree in Medicine, which subjects did you learn about regarding palliative care?

- Managing near-fatal symptoms
- Advanced care planning
- Living will
- Pain management
- Managing symptoms other than pain
- Palliative care service delivery system
- Providing care for families and caregivers

5. Have you ever received additional training on palliative care while studying in Medicine beyond what was provided in the institute such as workshops or meetings? If so,…….... times

**Part 2 Practice and Confidence ( 3 items )**

Direction: Please answer the questionnaire about yourself by ticking ( √ ) in front of the corresponded answer or fill in the blank.

1. The number of terminally ill patients have you treated in the past 3 months

- None
- Experienced, specify the number of .............. people

2. Are you confident in your skills to provide palliative care? ( 0 = least, 10 = most )

|  |  |  |  |  |  |  |  |  |  |
| --- | --- | --- | --- | --- | --- | --- | --- | --- | --- |
|  |  |  |  |  |  |  |  |  |  |

0 1 2 3 4 5 6 7 8 9 10

3. Are you confident in your skills to screen patients for referral to a palliative care medical team? ( 0 = least, 10 = most )

|  |  |  |  |  |  |  |  |  |  |
| --- | --- | --- | --- | --- | --- | --- | --- | --- | --- |
|  |  |  |  |  |  |  |  |  |  |

0 1 2 3 4 5 6 7 8 9 10

**Part 3** **Knowledge about palliative care ( 16 items )**

Directions: Choose the best answer by crossing ( X ) in front of the text that most closely matches your response.

1. Orawan, a 65-year-old woman, has been diagnosed with advanced cervical cancer. Her husband and son have requested you not to inform her this matter, assuming that she would not accept. As a palliative care doctor, how would you handle this situation?
2. Comply with her family wishes and tell her with only the necessary information about her disease
3. Consult with the family to determine what information should be given to Orawan, and provide her with that information while discarding any other details
4. Inform the family that it is the patient's right to know her relative information, and decline to withhold the prognosis from Orawan
5. Disclose Orawan's illness to her in a manner you feel is appropriate, while avoiding any information that could negatively affect her expectations"
6. Which is not the following reasons do you consider for referring Orawan to a palliative care unit?
7. To help Orawan and her caregivers in coping with mental health issues
8. To provide Orawan with alternative options if she prefers institutional treatment for terminal illness over receiving palliative care from you
9. To offer social services as part of outpatient treatment
10. To familiarize the palliative care team when a referral is required
11. A male patient with metastatic colon cancer comes to see you with back pain. As a palliative care doctor, you must treat the patient with painkillers. At what stage do you think opioids should be given for pain relief?
12. For all stages of cancer
13. When the prognosis is that the patient will live less than 1 year.
14. When the prognosis is that the patient will live less than 6 months.
15. When the prognosis is that the patient will live less than 3 months.
16. Suda, a breast cancer patient, is currently taking oral normal release tramadol at a dose of 50 mg every 4 hours for pain relief. Which of the following is the most appropriate sustained release tramadol dose for her?
17. 100mg sustained release tramadol every 12 hours
18. 200mg sustained release tramadol every 12 hours
19. 300mg sustained release tramadol every 12 hours
20. 300mg sustained release tramadol every 8 hours
21. To a patient who has been taking opioid painkillers for weeks and complaining of increasing pain, what do you think is the reason?
22. Developing drug tolerance
23. Drug dependence
24. The patient's disease is getting worse.
25. A state of drug addiction
26. Somsak, a 65-year-old man diagnosed with advanced lung cancer, is experiencing nausea and vomiting when given tramadol. Do you think it's appropriate to treat his nausea and vomiting by administering opioids, except....
27. Metoclopramide
28. Dexamethasone
29. Haloperidol
30. Dimenhydrinate
31. If Somsak, a 65-year-old man diagnosed with advanced lung cancer, has decreased kidney function and becomes confused while taking sustained release tramadol, Somsak's most appropriate treatment decision was....
32. Change the dosage form from oral to intramuscular injection
33. Give haloperidol as an additional treatment
34. Switch to normal tramadol form and adjust to the appropriate dose to reduce pain
35. Reduce the dose of sustained release tramadol and monitor the pain level
36. When considering the administration of strong opioids to a 45-year-old patient diagnosed with liver cancer and with normal bowel function, when is the appropriate time to administer a laxative to the patient?
37. At the same time as dispensing the opioids
38. When she has not been able to have a bowel movement normally for several days.
39. When she has not been able to have a bowel movement normally for more than a week
40. When she complains that her bowel movements are less than usual
41. During your Friday afternoon clinic, you met with Amporn, a patient with metastatic breast cancer. She expressed contentment with her current quality of life, even though her prognosis is only six months. However, she complained of back pain and numbness at the L5 level, along with difficulty urinating. You suspect spinal cord compression. In this situation, the most appropriate treatment for the patient is.......
42. Admit the patient to the hospital to prepare for an MRI.
43. Send the patient to the emergency department for X-rays.
44. Review the patient's history and make an appointment to recheck the patient's cord compression symptoms on Monday.
45. Refer the patient to an oncology ward or p alliative care for assessment
46. The key symptoms of hypercalcemia in cancer patients are:
47. Confusion, diarrhea and thirst
48. Nausea, constipation and confusion
49. Diarrhea, thirst and tinnitus
50. Nausea, tinnitus and dyspepsia
51. A 20-year-old male patient from Thailand has been diagnosed with advanced prostate cancer. He presented to you with weakness, extreme difficulty breathing, and symptoms unrelated to stress. You believe that focusing on breathing exercises will not improve the patient's condition. The appropriate treatment for the patient is....
52. Administer oxygen at home to keep O2 sat to the patient at 95% level.
53. Instruct the patient to use a paper bag for breathing exercises.
54. Administer immediate release opioids prior to PRN.
55. Arrange for the patient to undergo an urgent X-ray to assess for airway obstruction or pleural effusion.
56. Uraiwan is a 50-year-old female patient with terminal ovarian cancer and a very poor quality of life. She has refused to take food and water, and her husband is very stressed out about her decision. As the doctor treating her, you should....
57. Help reassure her husband and arranged for her husband to consult if he wishes.
58. Give Uraiwan fluids intravenously to help her get enough water.
59. Respect Uraiwan's wishes.
60. Give her sedation to make her feel more comfortable.
61. Uraiwan is being cared for at home during the last few days of her life, her family is understandably emotional about the situation. It would be useless to reassure the patient and her family. The following medications would be best suited for this purpose are:
62. Lorazepam
63. Morphine
64. Hyoscine hydrobromide
65. Haloperidol
66. You met with Uraiwan's husband two weeks after his wife's passing. What factors do you think could cause her husband to receive counseling after a severe bereavement..........
67. He could not abandon his wife's clothes.
68. He took pictures of her to look at every few hours
69. He was unable to adjust to even his son or his friends.
70. He starts to cry when you talk about his wife.
71. Regarding to living will, any item that the doctor cannot comply with
72. Do not want to press the heart massage if the heart stops beating.
73. Wants morphine to relieve pain.
74. Do not want a nasal feeding tube when oral intake is not possible.
75. Wishing for high-dose morphine to speed up the patient's peaceful death
76. Who is not allowed to make a living will?
77. Those who can't read
78. Those who have incomplete consciousness
79. A person who is healthy, does not have a disease
80. Minors under the age of 20 with parental consent.

**Part 4** **Attitude towards palliative care ( 22 items )**

Direction: Please indicate 1-5 level ( 1= strongly disagree, 2= disagree, 3= uncertain, 4= agree, 5= strongly agree ) by ticking ( √ ) in the box of your answer.

|  | Items | Strongly disagree  (1) | Dis-  agree  (2) | Uncer-  tain  (3) | Agree  (4) | Strongly agree  (5) |
| --- | --- | --- | --- | --- | --- | --- |
| 1 | I am satisfied with treating patients who are expected to recover than those who are expected to die. |  |  |  |  |  |
| 2 | Patients should not know how serious their illness is till the end of their lives. |  |  |  |  |  |
| 3 | If the patient talks about death, doctors and nurses should reassure them that it is not something to be worried about. |  |  |  |  |  |
| 4 | Patients with terminal cancer should be assessed less frequently than patients with early-stage disease. |  |  |  |  |  |
| 5 | Even if the patient's relatives do not ask any advices, the doctor should give advice about with the terminal stage cancer patients. |  |  |  |  |  |
| 6 | To treat a terminally ill cancer patient can remind doctors of their own feelings if they were terminally ill. |  |  |  |  |  |
| 7 | Relatives who are providing close care for terminally ill cancer patients to frequently interfere with the medical care provided. |  |  |  |  |  |
| 8 | If I could choose, I would not take care of terminally ill cancer patients. |  |  |  |  |  |
| 9 | Nurses are the primary healthcare professionals to deal with cancer patients at the initial stage. |  |  |  |  |  |
| 10 | It is essential for doctors to prepare cancer patients before they enter the final stages of the disease. |  |  |  |  |  |
| 11 | End-stage cancer patients should let their symptoms get worse on their own rather than help them have a longer quality of lives. |  |  |  |  |  |
| 12 | Doctors are an key person in helping to reduce suffering for terminally ill cancer patients. |  |  |  |  |  |
| 13 | Terminally ill cancer patients are discouraged by their disease because healthcare professionals express their discouragement in treating them. |  |  |  |  |  |
| 14 | I always feel comfortable when I discuss terminal cancer patients issue with other doctors as a team. |  |  |  |  |  |
| 15 | I wouldn't worry about a cancer patient's have drug addiction if my family members were taking morphine for pain relief. |  |  |  |  |  |
| 16 | It is an proper treatment for cancer patients to receive opioids for pain relief at all stages of the disease. |  |  |  |  |  |
| 17 | Terminally ill cancer patients who can still talk about the future work and/or travel Indicate that they do not feel that their disease is severe. |  |  |  |  |  |
| 18 | Most cancer patients can often feel overwhelmed and emotional as they near the end of their lives. |  |  |  |  |  |
| 19 | Doctors and nurses working with terminally ill cancer patients often have mantal issues. |  |  |  |  |  |
| 20 | Doctors should advise cancer patients to avoid getting angry when their symptoms are out of control. |  |  |  |  |  |
| 21 | I think advanced care planning with the patient and family ( advance care planning ) improves the quality of life for terminally ill patients. |  |  |  |  |  |
| 22 | Healthcare professionals should provide care for terminally ill patients according to living will. |  |  |  |  |  |

**Part 5** **Palliative care vs Euthanasia ( 8 items )**

Directions: Please Tick ( √ ) in the box “Palliative care” and/or “Euthanasia” that best represent the actions or intentions of the doctor described in the table.

|  | Detail | Palliative care | Euthanasia |
| --- | --- | --- | --- |
| 1 | Giving sedative drugs to terminal stage patients to reduce the patient's consciousness in the hope of relieving uncontrollable suffering |  |  |
| 2 | Slow morphine dose increase ( morphine titration ) to give terminal patients relief from breathlessness |  |  |
| 3 | Rapid overdose of morphine for terminally ill patients to die |  |  |
| 4 | Doctors do not intubate endotracheal tubes for end-stage cancer patients with respiratory failure because they want to comply with a living will. |  |  |
| 5 | Doctors allow relatives to take terminal stage patients to home according to the patient's wills. |  |  |
| 6 | Doctors give sleeping pills or muscle relaxants. with the intention of causing the patient to die as the patient wishes while fully conscious |  |  |
| 7 | Dr. Somchai is the medical specialist responsible for treating patients with terminal lung cancer. He has been providing them with surgery administration, chemotherapy, and irradiation. Despite his best efforts, the cancer continues to spread and the patient has become very tired and weak. Then he asked the patient's relatives to decide on a treatment plan that prioritizes the patient's comfort and quality of life, which may mean discontinuing chemotherapy and focusing on symptom management. |  |  |
| 8 | Dr. Supol uses Thiopental, an anesthetic, to speed up the patient's peaceful death according to the needs of terminally ill patients who cannot be cured |  |  |
